# Human red blood cell ATP content and export under hypoxic and/or isocapnic storage conditions

**DOI:** 10.3389/fphys.2025.1641343

**Published:** 2025-09-18

**Authors:** Youwei Chen, Cole Darrow, Aidan Murray, Thomas Wise, Zhong Lucas Li, Nisha Srivastava, Hongmei Zhu, Ian J. Welsby, Tatsuro Yoshida, Tim J. McMahon

**Affiliations:** ^1^ Department of Medicine, Duke University Health System, Durham, NC, United States; ^2^ Department of Medicine, Durham VA Health Care System, Durham, NC, United States; ^3^ Hemanext, LLC, Lexington, MA, United States

**Keywords:** erythrocyte, transfusion, oxygen binding, biphosphoglycerate (BPG), blood flow

## Abstract

**Introduction:**

In some diseases driving or associated with anemia, red blood cell (RBC) transfusion conveys limited benefit, and only when the anemia is severe. The banking of RBCs alters key molecules and functions. Among these changes during blood banking, depletion of the allosteric effector 2,3-BPG (biphosphoglycerate) takes place in the first two to 3 weeks, while ATP depletion only becomes prominent in the fifth or sixth (i.e., final) weeks of storage. One approach to testing the significance of these changes is to test the effects *in vitro* and *in vivo* of stabilizing key molecules. We hypothesized that hypoxic RBC storage, which can stabilize RBC BPG and ATP generation, could in turn stabilize the ability of RBCs to export vasoactive ATP, an activity that modulates RBC functions including O_2_ delivery.

**Methods:**

We performed a parallel study of conventional RBCs, hypoxically stored (Hemanext) RBCs (“HN-Std RBCs”), and CO_2_-preserved, hypoxically stored RBCs (“HN + CO_2_ RBCs”).

**Results and discussion:**

Standard hypoxic RBC storage boosted RBC ATP content, peaking in mid-storage. The time course of P_50_ (a measure of RBC Hb O_2_ affinity) changes in hypoxically stored RBCs corresponded to that of superior preservation of BPG, peaking in the first one to 2 weeks of storage. CO_2_-preserved hypoxic RBCs preserved ATP within the first week of storage, but with little effect on BPG or P_50_ at any time point. ATP export from RBCs assessed in normoxia or hypoxia declined over storage time, and generally did not differ significantly as a function of hypoxic storage ± CO_2_ preservation. An exception was the 1-week timepoint, when ATP export was significantly greater by HN + CO_2_ stored RBCs than by HN-Std stored RBCs. Taken together, these findings demonstrate time-dependent, differential modulation of RBC ATP and BPG by hypoxic RBC storage with or without CO_2_ preservation. Overall, hypoxic RBC storage ± CO_2_ preservation neither promotes nor restricts RBC ATP export to a large extent as compared to conventional RBC storage. Given that transfusion of hypoxically stored RBCs can be advantageous, future studies can test whether the differential and time-dependent effects on ATP, BPG and P_50_ can be leveraged for context-specific or personalized decision-making around RBC transfusion for anemia.

## 1 Background

Anemia, even when mild, is a well-established negative risk factor for poor outcomes in critical illness and other conditions. But paradoxically, RBC transfusion is beneficial only when anemia is severe (Wise et al., 2024). Taken together, these findings suggest that the function of RBCs stored for transfusion could be improved. Changes in RBC metabolites and functions during blood banking may play a role in these disappointing responses to transfusion. Interestingly, the timing of changes in key molecules is quite variable. For example, stored RBCs are brought to low-pH condition within hours; 2,3-biphosphoglycerate (2,3-BPG, aka BPG or 2,3-DPG), a key allosteric effector of RBC hemoglobin’s O_2_-offloading function, declines over the first 2 weeks; and declines in RBC ATP take place more gradually over the typical 6 weeks of storage (Bennett-Guerrero et al., 2007). The complex array of biochemical and functional changes and their timing may contribute to the general lack of benefit of transfusing “fresher (e.g., stored < 7 days) RBCs” (Tinmouth et al., 2006; Lacroix et al., 2015).

Hypoxic RBC storage was reported to slow oxidative lesions, preserve the organic phosphates ATP and BPG (Yoshida et al., 2019), and stabilize critical post-storage behaviors of RBCs (Burns et al., 2016; Yoshida et al., 2017). Cellular deformability is better maintained with hypoxic than conventional RBC storage, with better morphologic stability, reduced vesiculation (RBC microparticle formation) and lower susceptibility to lysis over time or in response to an osmotic stress (Yoshida et al., 2017). O_2_-unloading kinetics are demonstrably faster in hypoxically stored RBCs vs. conventionally stored RBCs (Rabcuka et al., 2022). In transfused rodents, RBCs stored hypoxically using the Hemanext method led to superior results in models of hemorrhagic shock with or without traumatic brain injury (Williams et al., 2020; Muller et al., 2024). In parallel, tissue markers of hypoxia and inflammation were attenuated, and left ventricular cardiac function was better preserved (Williams et al., 2020).

While hypoxic storage of RBCs appears to preserve intra-RBC ATP, its effects on the ability of RBCs to export ATP have not been determined. The ability of RBCs to export vasoactive mediators such as ATP plays a role in O_2_ delivery, given that RBCs can influence blood flow, which is the “second arm” of the O_2_ delivery formula (fractional O_2_ offloading being the first). Conventional storage depresses the ability of RBCs to export vasoactive ATP (Zhu et al., 2011), both basally and in response to physiological cues such as hypoxia (when RBC-dependent vasodilator reflexes act to increase regional blood flow and thus O_2_ delivery). We previously linked this depressed ATP export capacity to modest oxygen desaturation after RBC transfusion in a mouse model (Zhu et al., 2011).

We hypothesized that Hemanext/hypoxic storage of RBCs would promote superior ATP-export capacity basally and in response to post-storage deoxygenation. We compared conventionally stored RBCs to both standard hypoxic/Hemanext (designated “HN-Std”) RBCs and to hypoxic/Hemanext RBCs exposed to ∼5% CO_2_ during processing and storage (designated HN + CO_2_) RBCs. The rationale for studying the effects of such CO_2_ “preservation” is that RBC ATP content may be further augmented when a relatively isocapnic (5% CO_2_) environment is maintained during hypoxic storage. Specifically, RBC ATP values are lower after the loss of CO_2_ that accompanies the RBC “hyperventilation (CO_2_ loss)” that occurs in the O_2_-purging (and CO_2_-free) gas exposure used for hypoxic RBC preparation (Dumont et al., 2016). The CO_2_-dependence of RBC ATP production may involve modulation of BPG mutase activity in the RBC. Indeed, the increased RBC ATP resulting from CO_2_ preservation comes at the expense of some BPG (Dumont et al., 2016). Thus, co-modulation of CO_2_ (or not) during low-PO_2_ storage could allow differential modulation of BPG and cellular and exported ATP.

## 2 Methods

### 2.1 Acquisition, preparation, shipment, and storage of human RBC units and “subunits”

De-identified human RBCs, procured from Rhode Island Blood Center by the study sponsor Hemanext, were conventionally acquired into CP2D solution after written informed consent. The units were then processed (including leukofiltration) and banked in conventional RBC storage bags using AS-3 additive solution (Haemonetics, Braintree MA). Each bag contained about 300 mL of RBCs. Two RBC units, selected based only on mutual ABO- and Rh-compatibility, were pooled, then split into three “subunits” (∼200 mL each) intended to become conventional (Control), hypoxic/Hemanext (HN-Std), and hypoxic/isocapnic (HN + CO_2_) RBCs. HN + CO_2_ RBC preparation was intended to preserve CO_2_ at levels matching those in Control RBCs during 6 weeks of refrigerated (4 °C) storage. In total, n = 8 pooled RBC superunits (from 16 standard units) from healthy adult human donors were used. No data were collected on donor sex, age or other demographic variables for the purposes of the study. In the case of five of the superunits, initial production of the subunits was performed in Durham, NC, and in the remaining three superunits, production was performed in Lexington, MA followed by overnight shipping to Durham, NC. In both cases, collected whole blood was processed within 24 h.

### 2.2 Hemanext (HN-Std) and Hemanext + CO_2_ (HN + CO_2_) RBC production

Hemanext ONE® hypoxic blood storage bags were provided by Hemanext Inc. (Lexington, MA). Standard Hemanext oxygen reduction was performed by following the Hemanext ONE instructions and using a Hemanext oxygen reduction bag (ORB). Meanwhile, the CO_2_-augmented (hereafter also referred to as CO_2_-preserved, HN + CO_2_) subunit was prepared by continuously supplying 5% CO_2_/balance nitrogen) gas mix in the space between RBC-containing bag and outer gas barrier bag and was otherwise handled identically to the standard Hemanext subunit. In order to achieve the target SO_2_ of 10% or lower, an estimate was made of the deoxygenation rate by calculating the rate of change in SO_2_ at an early time point (5–10 min) and a later time point (60–90 min) of the Hemanext deoxygenation process. The RBC concentrates from the ORBs were then transferred into respective (HN-Std and HN + CO_2_) Hemanext storage bags (PVC bag enclosed by gas barrier bag with sachet of O_2_/CO_2_ sorbent placed between the inner and outer bag), and placed in a 4 °C cold room. The Control subunit was exposed to no gas, stored in a conventional PVC RBC bag exposed to room air, and otherwise handled identically.

On each study day [on Days 3 ± 1 (one of the 7 assay sets was run on both day 2 and day 4; one of the seven on Day 2 only; and the other five on Day 3), 7–8 (2 of 8 on Day 8), Day 14, Day 21 and 42–45 (one of 8 on day 45) after acquisition] a small aliquot was removed anaerobically from each subunit bag. For simplicity, we refer hereafter to these timepoint ranges as Days 3, 7, 14, 21, and 42. (The averaged value of assay results from Days 2 and 4 (both from the same source) was imputed as a Day 3 value in one case, and a Day 45 value was imputed as Day 42). Other than brief, airtight sampling at these intervals, the units were stored conventionally at 4 °C throughout the 42-day maximal storage duration.

At each study timepoint, subunit RBC aliquots (12–17 mL) were withdrawn from the storage bag after gentle resuspension of the RBCs. Subaliquots were set aside before (“subunit suspension”) and after separation by centrifugation at 2700 RPM at 4 °C for 3 min (yielding a “subunit supernatant’ and a ‘pellet’). The RBC suspension samples were immediately transported to a cooximeter/blood gas analyzer (GEM Premier 5000, Werfen, Spain) operated by Duke University Hospital Clinical Laboratories (see below). Remainder subunit suspension samples were lysed hypotonically as described (Kirby et al., 2021), and assayed for hemoglobin (Hb) and ATP content (Kirby et al., 2021). RBCs were resuspended in Krebs-Henseleit buffer at 1% Hct and kept on ice in preparation for gas exposure (“tonometry”). This was repeated for each sample from each subunit on each day of the serial assays.

### 2.3 O_2_ Dissociation curve (ODC) construction and P_50_ calculation

After completion of co-oximetry, the RBC suspension was transported in a syringe on ice for processing utilizing a Hemox Analyzer (TCS Scientific). Following manufacturer guidelines, 20 µL of bovine serum albumin 20% (BSA-20) and 10 µL of anti-foaming agent (TCS) were added to a microtube containing 5.0 mL of Hemox buffer solution (composition: 30 mM N-Tris (hydroxymethyl)methyl-2-aminoethanesulfonic acid (TES), 130 mM NaCl, and 5 mM KCl; pH 7.40 ± 0.01). Finally, 50 µL of RCC (red cell concentrate) from each sample was pipetted into the corresponding microtube, and capped microtubes were refrigerated at 4 °C until ODC analysis. This process was repeated for aliquoted samples from each subunit (Control, HN-Std, and HN + CO_2_).

Using the Hemox Analyzer, 3 mL of the blood-buffer solution was added to the sampling cuvette and warmed until the temperature was steady at 37 °C. While incubating, the samples were fully oxygenated using medical air (21% O_2_) until the Hemox Analyzer oxygen sensor demonstrated a steady partial pressure of oxygen (PO_2_). Once the blood-buffer suspension was fully oxygenated and stable at 37 °C, the PO_2_ was manually set to 148 mmHg to represent the calculated (predicted) partial pressure of O_2_ (PO_2_) in room air given the atmospheric pressure and elevation of our laboratory site in Durham, NC. The sample was then progressively de-oxygenated by switching the gas supply from air to nitrogen (N_2_) gas. Utilizing the OEC3 Software (TSC Scientific), real-time data on PO_2_ and SO_2_ (percent hemoglobin saturation by O_2,_ measured directly by dual wavelength spectrophotometry) during the deoxygenation process was obtained. Once each sample was adequately deoxygenated (PO_2_ < 1 mmHg), the resulting oxygen-dissociation curve (ODC) was displayed and stored, and the P_50_ was calculated by the OEC3 software. Hemox utilizes spectrophotometry to compare absorption of oxyhemoglobin and deoxyhemoglobin, with ratios calculated by OEC3 software to generate ODCs. The sample cuvette was then washed with de-ionized water three times before loading the next RBC-buffer sample for processing. This analytical method helps ascribe differences in ODCs to differences in intracellular factors, because a constant temperature and pH are maintained throughout the oxygenation and deoxygenation process.

### 2.4 RBC exposure to controlled normoxia or hypoxia for export assay

In order to determine O_2_-sensitive RBC ATP export, the RBC samples (in Krebs buffer, pH 7.40) at 1% hematocrit were subaliquotted and placed in a rotating-bulb tonometer at 37 °C. To establish hypoxic or normoxic conditions in the rotating tonometer, a gas blender was used to establish “hypoxia” (94% N_2_, 1% O_2_, 5% CO_2_) and “normoxia” (74% N_2_, 21% O_2_, 5% CO_2_) conditions at 200 mL/min gas flow (in total to two parallel tonometers) over thin-film RBC suspensions (not “bubbled through”). 1.5 mL of each sample was added via a port on the tonometer before re-sealing, and was then exposed gently to gas for 8 min, sufficient for reaching steady-state changes in oxygenation, as previously described (Kirby et al., 2012). Sample exposures were performed in duplicate.

Following gas exposure, the post-tonometry sample suspension was centrifuged at 2,700 RPM at 4 °C for 3 min. The resulting supernatant was removed for exported ATP and free Hb assays. In the event that Hb and ATP assays could not be performed on the same day as the gas exposure, the corresponding samples were stored at −20 °C. We have previously determined that the values for both analytes (which are acellular) do not change with 1 or 2 freeze-thaw cycles. Subunit suspension samples (10 μL) were diluted in two steps (total 1000-fold dilution) into deionized water, then vortexed to produce a lysate for assays of total intracellular Hb and ATP. The Hb value is used as the “denominator” in RBC lysis calculations post tonometry.

### 2.5 Hemoglobin and ATP assays

Free (supernatant) and intracellular Hb values for each sample were obtained using a spectroscopic method via a microplate reader as described (Zhu et al., 2011). For ATP assays, the luciferin-luciferase assay was used, and values were extrapolated against an ATP standard curve generated daily in PBS (as described) (Kirby et al., 2021).

### 2.6 2,3-BPG assay by LC-MS/MS

Red blood cells (10 µL) were mixed with 200 µL methanol and 10 µL 10 μg/mL ^13^C_3_-2,3-BPG. The resulting supernatant samples were subjected to complete dryness under nitrogen, then resuspended into 80 µL 80% methanol solution before instrument injection. Samples were analyzed with the Sciex QTrap 6500+ system (Framingham, MA) with Waters Acquity I-class plus UPLC. Software Analyst 1.7.3 was used for data acquisition. The LC separation was performed on a Waters Acquity UPLC Phenyl-Hexyl column (2.1 × 100 mm, 1.7 μm) with mobile phase A (20 mM ammonium acetate with 0.02% ammonium hydroxide in water) and mobile phase B (acetonitrile). The flow rate was 0.45 mL/min. The linear gradient was as follows: 0–1 min, 0%B; 1–2.9 min, 50%B; 3–4.5 min, 95%B; 4.6–5.1 min, 0%B. The autosampler was set at 10 °C and the column was kept at 40 °C. The injection volume was 5 μL. Mass spectra were acquired under negative electrospray ionization (ESI) with the ion spray voltage of −4500 V. The source temperature was 450 °C. The curtain gas, ion source gas 1, and ion source gas 2 were 35, 50, and 70 psi, respectively. Multiple reaction monitoring (MRM) was used for quantitation as shown below: 2,3-BPG (target): m/z 265.1 → m/z 79.0; 13C_3_-2,3-BPG (internal standard): m/z 268.1 → m/z 79.0.

### 2.7 Blood gas and Co-Oximetry measurement

RBC samples were transported immediately on ice to the co-oximetry laboratory. Utilizing a GEM Premier 5000 arterial blood gas analyzer (Werfen), ∼150 µL of sample was aspirated from each syringe for analysis before re-capping the syringe and returning it to ice. Co-oximetry results generated by the GEM Premier 5000 analyzer included concentration of total hemoglobin (tHb), oxyhemoglobin (oxyHb), carbonmonoxyhemoglobin (COHb), methemoglobin (metHb), and oxyhemoglobin saturation (SO_2_). Additional results obtained include pH, PCO_2_, and PO_2_. All were measured at 37 °C.

### 2.8 Statistical analyses

All values are reported as individual data and the mean ± SEM unless otherwise noted. In the case of missing values, there was no imputation. Testing across assay exposure (performed in normoxia or hypoxia), RBC “treatment” status, and over time was performed using two-way repeated measures ANOVA mixed-effects model with appropriate *post hoc* sample testing corrected for multiplicity (Tukey’s). Statistics were performed using GraphPad Prism 10. *p* < 0.05 (*) was considered statistically significant unless otherwise noted. ** indicates *p* < 0.01; *** indicates *p* < 0.001; ****p < 0.0001.

## 3 Results

### 3.1 Blood gases, cooximetry, and pH

**FIGURE 1:**
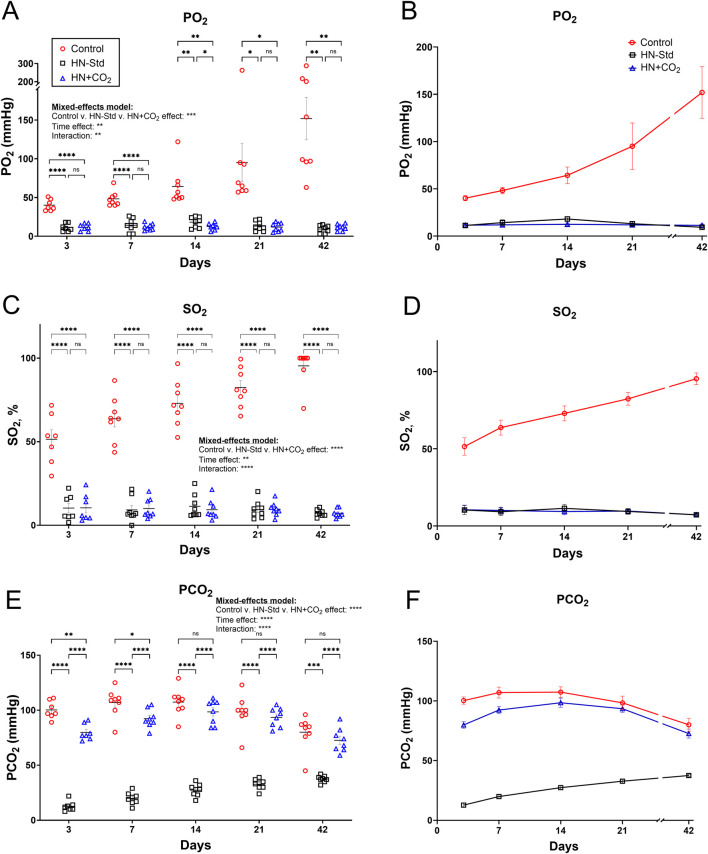
PO_2_, SO_2_, and PCO_2_ over time during storage of matched control/conventionally stored RBCs (Control), HN-Std (standard hypoxic Hemanext) RBCs; and HN + CO_2_ (CO_2_-preserved hypoxic Hemanext) RBCs. **(A,B)** PO_2_ during RBC storage. From Day 3 to Day 42, the PO_2_ levels in both HN-Std and HN + CO_2_ were significantly lower than in Control RBCs (*p* value levels: *, *p* < 0.05; **, *p* < 0.01; ***, *p* < 0.001; ****, *p* < 0.0001). PO_2_ did not differ significantly between HN-Std and HN + CO_2_ RBCs. **(C,D)** SO_2_ in both HN-Std and HN + CO_2_ were significantly lower vs. Control (*p* < 0.0001 at each time point from Day 3 to Day 42). SO_2_ in HN-Std RBCs did not differ significantly vs. HN + CO_2_ RBCs. **(E,F)** PCO_2_ during RBC storage. In HN-Std RBCs, PCO_2_ levels were significantly lower at each timepoint during RBC storage (*p* < 0.0001 vs. Control and vs. HN + CO_2_), as expected. PCO_2_ did not differ significantly between HN + CO_2_ RBCs and Control RBCs overall (by mixed-effects analysis), but at Days 3 and 7 the small differences (via *post hoc* t-tests) were statistically significant. For visual clarity, identical data are shown in the left-sided panels (individual values, mean ± SEM; **(A,C,E)**) and the right-sided panels (**(B,D,F)**; showing mean ± SEM and non-fitted connector lines). The statistical results are shown in the left panels only. N = 8 except 7 on Day 3.

Blood gas and cooximetric assays of 4 parent superunit pools (3 subsets each) of standard HN-Std and HN + CO_2_ RBCs confirmed that their actual SO_2_ values were near 10% (Supplementary Figure S1), in line with the projected SO_2_ target at 10% based on a rate calculation made from SO_2_ change during the process. Figures 1A,B, shows that while PO_2_ levels in the Control (conventional) RBC subunit gradually increased over storage time, the PO_2_ in both HN-Std and HN + CO_2_ RBC subunits were initially and remained much lower. PO_2_ differences were significant from Day 3 to Day 42 (*p* values ranged from <0.05 to <0.0001). The SO_2_ data were similar to the PO_2_ data: significantly lower in the HN-Std and HN + CO_2_ RBC groups vs. the Control group (*p* < 0.0001) at every sampling time point during RBC storage (Figures 1C,D). Notably, SO2 values in the HN-Std and HN + CO_2_ RBCs were near 10% on Day 3, indicating stability of the initial deoxygenation effect. When comparing the two (HN + CO_2_ vs. HN-Std) Hemanext RBC subunits, there were generally no statistically significant differences on any sampling day for either PO_2_ or SO_2_. PCO_2_ levels in the CO_2_-preserved RBC subunit exceeded those in the standard Hemanext (*p* < 0.0001) RBCs. PCO_2_ values in the HN + CO_2_ subunits were modestly but significantly lower than in the Control subunits on Day 3 (*p* < 0.01) and Day 7 (*p* < 0.05), then matched those in the conventional subunit across all sampling time points from Day 14 to Day 42 of RBC storage (not significant (n.s.); Figures 1E,F). Hypoxic RBC preparation and storage, with or without CO_2_ augmentation, did not generate substantial excess methemoglobin (oxidized Hb; Supplementary Figure S2). pH values (not shown) in nearly all samples at all time points were reported as “< 6.93,” i.e., outside of the GEM Premier 5000 analyzer’s clinical reportable range for pH (6.93–7.72). The only exceptions were that in <5% of cases of PO_2_ measurements, instrument errors were reported; these error reports were evenly distributed with respect to RBC condition and storage time.

### 3.2 Changes in ODCs and P_50_ in RBCs stored hypoxically (+/− CO_2_ preservation)

**FIGURE 2:**
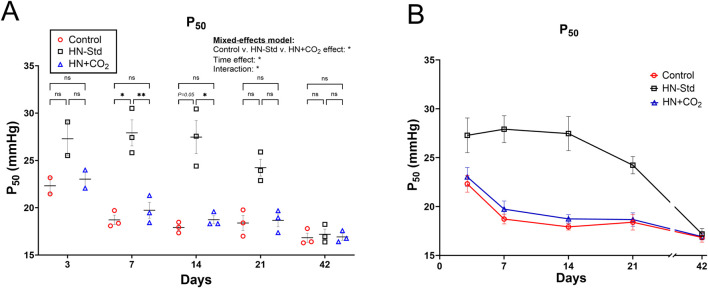
P_50_ measurements from three RBC types over time during RBC storage. Control, conventional RBC storage; HN-Std, standard Hemanext (hypoxic) RBC storage; and HN + CO_2_, CO_2_-augmented Hemanext (hypoxic) RBC storage. P_50_s for HN-Std RBCs vs. HN + CO_2_ or vs. Control groups differed statistically significantly by mixed-effects analysis where indicated (either *p < 0.05, or **p < 0.01). The HN + CO_2_ and Control subunits did not differ significantly from one another at any time point. For visual clarity, identical data are shown in the left-sided panel (individual values, mean ± SEM, **(A)** and in panel **(B)**, showing mean ± SEM and connector lines; not fitted). The statistical results are shown in **(A)** only. N = 3 except 2 on Day 3.

We observed changes in the ODCs (Supplementary Figure S3) and P_50_ (Figure 2) throughout the storage period that varied across the three RBC storage conditions. ODCs were shifted rightward (Supplementary Figure S3) and the P_50_ (Figure 2) was elevated (i.e., O_2_ affinity was decreased) in the HN-Std group compared to the HN + CO_2_ and Control groups except at Day 42. By mixed-effects analysis (Figure 2), storage condition-wise differences in P_50_ were statistically significant overall, and by *post hoc* testing at Days 7 and 14. By Day 42 there was no difference in mean P_50_ (*p* = 0.876). The higher mean P_50_ in the HN-Std RBCs over at least the first 3 weeks of storage implies a higher capacity for oxygen dissociation (offloading). These findings demonstrate that during standard hypoxic RBC storage (HN-Std), a physiologic P_50_ is approached, but this is not the case in CO_2_-preserved/hypoxic (HN + CO_2_) or Control RBC subunits (in both of which P_50_ is below that in normal healthy blood).

### 3.3 Intra-RBC ATP during hypoxic (+/− preserved-CO_2_) vs. control RBC storage

**FIGURE 3:**
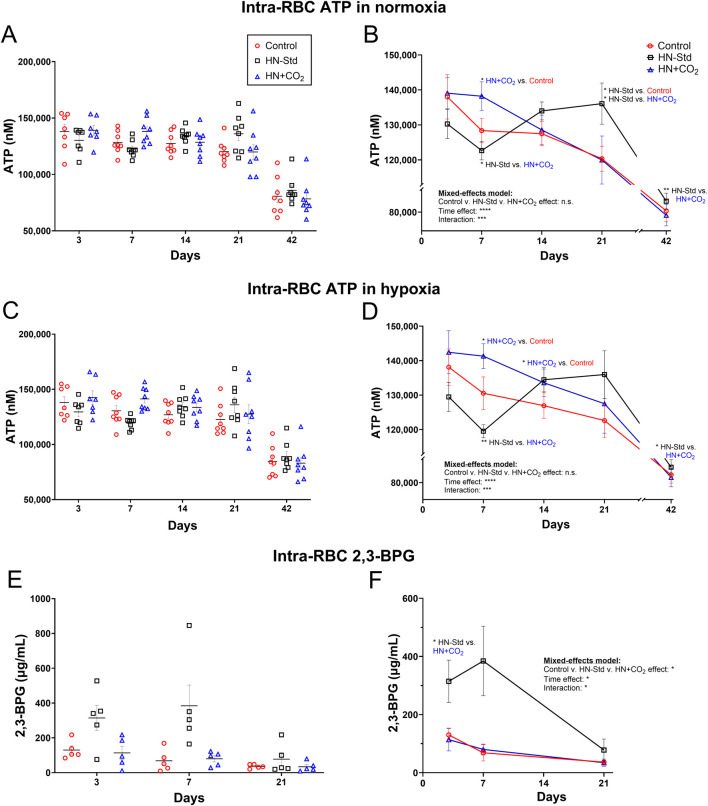
RBC intracellular ATP levels assayed after tonometry using normoxia **(A,B)** or hypoxia **(C,D)** in all three RBC types sampled over time during RBC storage. Control, conventional RBC storage; HN-Std, standard Hemanext RBC storage; and HN + CO_2_, CO_2_-augmented Hemanext RBC storage. The intra-RBC ATP levels in the Control subunits dropped significantly over storage, as assayed via both normoxia and hypoxia exposures. HN-Std intra-RBC ATP rose on Days 14 and 21 (*p* < 0.05 for normoxia), compared to that in Control RBCs. On Day 7, ATP levels in HN + CO2 RBCs were higher (*p* < 0.05 for normoxia; and *p* < 0.01 for hypoxia) vs. in HN-Std RBCs, but were lower than HN-Std on Day 21 (*p* < 0.05 for normoxia). By Day 42, ATP in all 3 RBC types was markedly depressed, but RBC ATP in the HN-Std units was significantly higher at Day 42 than in the HN + CO_2_ subunits. 2,3-BPG levels on Days 3 and 7 of RBC storage were greater (*p* < 0.05) in HN-Std RBCs than in HN + CO_2_ or Control RBCs. By Day 21, 2,3-BPG was markedly depressed in all RBC types **(E,F)**. 2,3-BPG levels were low and stable in, and did not differ significantly between, HN + CO_2_ and Control RBCs during storage. For visual clarity, identical data are shown in the left-sided panels (individual values, mean ± SEM, **(A,C,E)** and the right-sided panels **(B,D,F)** (showing mean ± SEM and non-fitted connector lines). The statistical results are shown in the right panels only. N = 8 at each time point except 7 on Day 3 for ATP and N = 5 at each time point for 2,3-BPG.

Intra-RBC ATP values declined between 21 and 42 days of storage (Figure 3). Standard hypoxic (HN-Std) storage tended to depress RBC ATP from Day 3 to Day 7, then to increase RBC ATP after Day 7, peaking at Day 21. Conversely, in HN + CO_2_ RBCs, intra-RBC ATP was stable from Day 3 to Day 7, then declined progressively through Day 42 (Figure 3). The changes in the intra-RBC ATP from Day 7 to Day 21 (a storage period during which RBCs are frequently transfused) therefore moved in opposite directions when comparing HN-Std (rising RBC ATP) and HN + CO_2_ (falling RBC ATP) RBC subunits (Figure 3). By Day 42, ATP in all 3 RBC types was markedly depressed, despite HN-Std still showing significant higher ATP level compared to the HN + CO_2_ subunit. Notably, we assayed for intra-RBC ATP in RBCs just after they had been exposed to normoxic (Figures 3A,B) or hypoxic (Figures 3C,D) gas for the ATP-export studies. The fact that RBC ATP values matched closely at each timepoint and under all conditions provides reassurance that the tonometry exposures did not substantially alter intra-RBC ATP by driving a large fraction into the extracellular space (note that extracellular ATP concentrations were >1000-fold lower than intra-RBC ATP).

### 3.4 Intra-RBC 2,3-BPG during hypoxic (+/− preserved-CO_2_) vs. control RBC storage

2,3-BPG levels on Days 3 and 7 of RBC storage were greater (*p* < 0.05) in HN-Std RBCs than in HN + CO_2_ or Control RBCs. By Day 21, 2,3-BPG was markedly depressed in all RBC types (Figures 3E,F). 2,3-BPG levels were stable in, and did not differ between, HN + CO_2_ and Control RBCs during storage.

### 3.5 RBC export of ATP during hypoxic (+/− preserved-CO_2_) vs. control RBC storage

**FIGURE 4:**
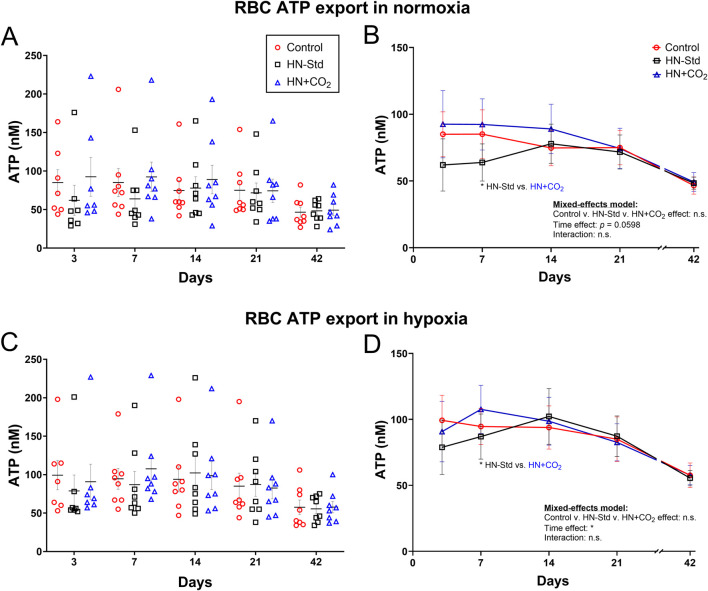
RBC ATP export assayed using tonometry in normoxia (upper panels **(A,B)**) or hypoxia (lower panels **(C,D)**) in three RBC types over time during RBC storage. Control, conventional RBC storage; HN-Std, standard Hemanext RBC storage; and HN + CO_2_, CO_2_-augmented Hemanext RBC storage. Other than the time effect, no other significant differences were found by 2-way ANOVA mixed-effects model (no group-wise differences), or by Tukey’s *post hoc* t-test at any timepoint except Day 7. For visual clarity, identical data are shown in the left-sided panels (individual values, mean ± SEM; **(A,C)**) and the right-sided panels (**(B,D)**, showing mean ± SEM and non-fitted connector lines). The statistical results are shown in the right panels only. N = 8 except 7 on Day 3.

Changes in RBC ATP export as a function of time, HN ± CO_2_ storage conditions, and normoxic or hypoxic assay conditions are displayed in Figure 4. Conventional RBC storage progressively depressed the ability of RBCs to export ATP in both normoxia and hypoxia (Figure 4), as we previously reported (Zhu et al., 2011). The effect of time was significant overall across the RBC preparation/storage conditions (*p* < 0.05 for hypoxia and *p* = 0.0598 for normoxia). The trend toward slightly greater RBC ATP export by HN + CO_2_ (CO_2_-augmented Hemanext) RBCs, and the downward trend in ATP export early during storage from HN-Std RBCs, reached statistical significance only on Day 7 in both normoxia (Figure 4A,B) and hypoxia (Figure 4C,D). In Control RBCs, intra-RBC ATP (which declined over the 6 weeks of storage) correlated inversely (*p* < 0.0001) with SO_2_, which rose over the 6 weeks of storage (Supplementary Figures S4A,B). By contrast, the decline in RBC ATP export did not correlate significantly with SO_2_ over time in Control RBCs (Supplementary Figures S4C,D). ATP export was significantly greater when assayed in hypoxia than in normoxia for conventionally stored RBCs, as previously reported (Zhu et al., 2011). ATP export was also significantly greater when assayed in hypoxia than in normoxia for HN-Std RBCs, but the difference did not reach statistical significance for HN + CO_2_ RBCs (Supplementary Figure S5).

### 3.6 [Hb] and RBC hemolysis in stored control, HN-Std, and HN + CO_2_ RBC subunits

Absolute Hb concentrations and calculated hemolysis values in the supernatants of all three actual RBC storage types, assayed serially over RBC storage time (Supplementary Figure S6) under varying O_2_ and CO_2_ conditions, did not differ significantly overall by mixed-effects analysis, nor were there significant differences by *post hoc* Tukey’s t-test at any timepoint.

### 3.7 RBC lysis in RBC suspensions after tonometry

**FIGURE 5:**
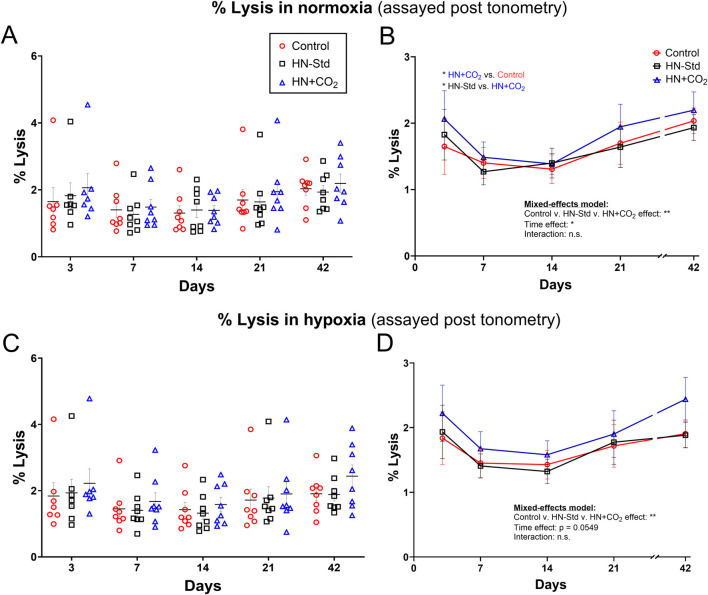
Post-tonometry RBC lysis in normoxia **(A,B)** or hypoxia **(C,D)** in three RBC types over time during RBC storage. Control, conventional RBC storage; HN-Std, standard Hemanext (hypoxic) RBC storage; HN + CO_2_, CO_2_-augmented Hemanext (hypoxic) RBC storage. Except at Day 3 for normoxia, there were no significant differences at any timepoint by *post hoc* Tukey’s t-test. For visual clarity, identical data are shown in the left-sided panels (individual values, mean ± SEM, **(A,C)**), and the right-sided panels **(B,D)** show the means ± SEM connected (but not fitted) over time. Statistical results are shown in the right panels only. N = 8 except 7 on Day 3.

RBC lysis was measured at the end of tonometric exposure to normoxic or hypoxic gas and expressed as a function of time and the O_2_/CO_2_ conditions during RBC storage (Figure 5). The effect of time was significant or nearly so (*p* < 0.05 for normoxia, and *p* = 0.0549 for hypoxia), and the changes were biphasic (U-shaped curve) under all three conditions, with an initial decline (visible for all 3 condition sets on Days 7 and 14). However, except on Day 3 for normoxia there were no significant differences at any timepoint by *post hoc* Tukey’s t-test. A fraction of the extracellular ATP we measured in tonometers could have resulted, at least in part, from this RBC lysis. When exported ATP in normoxia was expressed as its ratio to % RBC lysis, values for this ratio followed an inverted U-shaped curve and were greatest around Day 14 for HN-Std and HN + CO_2_ RBCs, but fell monotonically for Control RBCs (Supplementary Figure S7). These trends suggest that the extent of hemolysis is only one determinant of extracellular ATP.

## 4 Discussion

We investigated the effects of hypoxic RBC storage, with or without CO_2_ preservation, on intra-erythrocytic ATP and the ability of RBCs to export ATP, both basally and in hypoxia as a function of RBC storage time. Key findings include that when CO_2_ preservation is incorporated into the hypoxic RBC storage scheme, RBC ATP levels at Day 7 of storage were superior (*p* < 0.05) when assayed either in normoxia or hypoxia. Interestingly, by 21 days of storage, the ATP levels in CO_2_-preserved hypoxic RBCs had declined, similar to Control RBCs, whereas ATP in RBCs stored under hypoxia but without CO_2_ preservation had rebounded and exceeded (*p* < 0.05 in normoxia; trend n.s. in hypoxia) that in the HN-Std RBCs. RBC CO_2_ can modulate BPG mutase, which is involved in converting BPG to an ATP precursor. Accordingly, these findings are consistent with increased ATP generation early in storage when CO_2_ is clamped at levels matching those in Control RBCs, but a delayed surge in ATP production when CO_2_ levels are low within hypoxic stored RBCs. This later ATP surge (around the 21-day midpoint of RBC storage) in standard (low-CO_2_) hypoxic RBCs could reflect ATP generation at the expense of BPG, which was abundant early in standard hypoxic (Hemanext) RBCs.

As in prior reports, hypoxic RBC storage without CO_2_ augmentation preserved BPG for at least the first 7 days of storage (D'Alessandro et al., 2020; D'Alessandro et al., 2023). Moreover, the trajectory of changes in the P_50_s and ODCs of these RBC groups was directionally consistent with the changes we measured and that others have reported in the negative allosteric effector 2,3-BPG in conventional and Hemanext stored RBCs (D'Alessandro et al., 2020; D'Alessandro et al., 2023). Specifically, the P_50_ in standard Hemanext RBCs was elevated (in a range similar to that in healthy blood) and steady during early storage timepoints (Days 7 and 14), whereas P_50_ fell in conventionally stored (Control) RBCs during this period. Such declines in BPG are well known to take place in Control RBC storage conditions, typically within the first two to 3 weeks. The trend in P_50_ in HN + CO_2_ RBCs followed that in Control RBCs, falling over the first 2 weeks. Together, these findings may be driven by the apparent ability of hypoxic RBC storage to slow the depletion of intracellular BPG, while preservation of CO_2_ promotes the depletion of intracellular 2,3-BPG (in favor of ATP, see below). Notably, we did not clamp the PCO_2_ or add any CO_2_ to samples during Hemox assays. However, this lack of adding or clamping CO_2_ during ODC assays applied to all sample types and timepoints, and therefore would not account for the ODC and P_50_ differences we observed.

The directionally different changes over storage time in RBC ATP and BPG as a function of CO_2_ preservation raise interesting possibilities. From a scientific perspective, this approach could be used to disentangle the effects of these functionally distinct organic phosphates, where ATP acts as an energy source and BPG as a modulator of the allosteric control of O_2_ binding and release by hemoglobin. One major alternative approach capable of augmenting organic phosphates, known as rejuvenation, uses “PhIPA” (a mixture of phosphate, inosine, pyruvate, and adenine; commercially available previously as Rejuvesol) to regenerate both ATP and BPG in stored RBCs; both are increased. Several modifications of storage conditions (including additive solution content) show potential for improving the quality of the RBC product (Anastasiadi et al., 2024). From a clinical applications perspective, the differential and time-dependent preservation or augmentation of ATP and BPG via hypoxic RBC storage (±CO_2_ preservation) could be of utility in developing and testing disease-specific or even personalized approaches to decision-making and management of anemic patients requiring RBC transfusion. Anemic patients do vary vastly in terms of O_2_ delivery constraints, O_2_ uptake efficiency (chronic and acute lung function), vulnerability to sequelae of hemolysis, and more, as a result of the broad range of conditions driving anemia.

RBCs export ATP both basally and in response to stimuli including hypoxia, deformation/shear stress, and hormones (Dietrich et al., 2000; Kirby et al., 2021; Nayak et al., 2024; Wise et al., 2024). Dysregulated RBC ATP export may play a role in conditions and diseases ranging from the disappointing clinical outcomes after banked-RBC transfusion to atherosclerosis (Alcicek et al., 2024; Zhang et al., 2025). We previously showed that RBC storage depresses the ability of RBC to export ATP basally and in hypoxia, reflecting in part the declines over time in intra-RBC ATP. These changes were confirmed in the present study. Contrary to our predictions, and despite the storage condition- and time-dependent differences in intra-RBC ATP preservation or generation, hypoxic RBC storage generally did not significantly alter the ability of RBCs to export ATP, either basally or in hypoxia. This lack of a major effect on ATP export (or its decline) was consistent irrespective of CO_2_ preservation. Notably, levels of exported ATP were low under all storage and assay conditions and throughout the storage period, consistent with prior reports from our group and others. Overall, ATP export was modestly but significantly higher in hypoxia than basally in normoxia, but in HN + CO_2_ RBCs this difference was smaller and did not reach statistical significance (Supplementary Figure S5). The lack of a large increase in ATP export capacity is an important safety signal, because excessive ATP fluxes can promote inflammatory signaling, including excessive leukocyte adhesivity (Cekic and Linden, 2016; Le et al., 2019). In Control RBCs, RBC ATP content and export correlated inversely during storage with the progressive increases in SO_2_, but the observation that ATP export also decreased over storage time under hypoxic/Hemanext conditions argues against increased storage SO_2_ accounting for the decreases in ATP export. The causes of the overall declines in RBC ATP export are likely multifactorial.

The interpretation of the changes in extracellular ATP as a function of hypoxic storage with or without added CO_2_ is confounded by the presence of significant RBC lysis. It is impossible to parse the precise extent to which the measured extracellular ATP (ATP_ec_) is a direct result of the concomitant RBC lysis, but the possibility that a majority of the ATP_ec_ is hemolytic in origin cannot be excluded. The degree of hemolysis after sample exposure to normoxic or hypoxic gas in these experiments was substantial (∼1–2.5%). We observed a significant rank order for RBC lysis at Day 3 in which HN-Std RBC lysis exceeded that in HN + CO_2_ RBCs, which exceeded that in Control RBCs. But at other time points and for the overall curve, RBC lysis generally did not differ significantly between the two assay PO_2_ settings (normoxic vs. hypoxic) or as a function of the O_2_ and CO_2_ storage conditions (Control vs. HN-Std vs. HN + CO_2_). This modest degree of RBC lysis would be predicted to elevate ATP_ec_ substantially. Taking into account the intra-RBC ATP content, assay hematocrit, and % lysis we estimate that maximally ∼25% of the extracellular ATP could be post-lytic (non-exported) in origin. This, in turn, could have blunted or masked modest differences in ATP export by RBCs from different experimental (O_2_/CO_2_) groups, storage durations, or assay conditions (normoxia vs. hypoxia). The reason for the elevated post-exposure RBC lysis is uncertain, but notably, RBC bag supernatant ATP_ec_ was very low (not shown) at each timepoint during RBC storage under all three condition sets (Supplementary Figure S6). Controlled exposure of RBCs to normoxic or hypoxic (±CO_2_ clamp) gas using a tonometer, while gentle, could be viewed as an RBC product “stress test,” and might predict post-transfusion intravascular or extravascular hemolysis, post-transfusion RBC survival, or other favorable and unfavorable consequences of RBC transfusion. Notably, ATP export declined at later points during RBC storage under all conditions in spite of progressive increases in assay-associated RBC lysis, indicating that these changes are not likely to be mechanistically linked. Indeed, the ratios of extracellular ATP to % RBC lysis followed an inverted U-shaped curve in the cases of HN-Std and HN + CO_2_ RBCs (but not Control RBCs), and were greatest around Days 7 and 14. These trends suggest that the extent of hemolysis is only one determinant of extracellular ATP, particularly where hypoxic RBC storage is concerned.

We found that apparent ATP export from conventionally stored RBCs declined progressively over the six-week storage period, in agreement with our prior published results. We found that hypoxic storage of RBCs, when combined with CO_2_ preservation, significantly improved ATP export assayed on Day 7 in both normoxia and hypoxia, as compared to results in HN-Std RBCs without CO_2_ preservation. Notably, on Day 7 HN + CO_2_ RBCs also had intracellular ATP levels higher than in the other two groups. At all other time points, however, and in the overall mixed-effects analysis, ATP export did not vary according to RBC storage conditions (±hypoxia ± CO_2_). The lack of difference in ATP export as a function of RBC storage conditions was also seen on Day 21, when standard HN-Std RBCs had intracellular ATP values greater than those in the Control or HN + CO_2_ groups. Our findings provide reassurance that in spite of varying, and at times superior, intra-RBC ATP maintenance, hypoxic storage of RBCs with or without CO_2_ augmentation does not promote excessive ATP export, which could be injurious given its role in inflammatory cascades.

## Data Availability

The raw data supporting the conclusions of this article will be made available by the authors, without undue reservation.
