# Supplementary material for: Human red blood cell ATP content and export under hypoxic and/or isocapnic storage conditions
Source: Front Physiol. 2025 Sep 18;16:1641343. doi: 10.3389/fphys.2025.1641343 (PMC12488716; doi:10.3389/fphys.2025.1641343)
Supplement: Supplementary file 1 [file DataSheet1.pdf]

Supplementary Material

Supplementary Figures

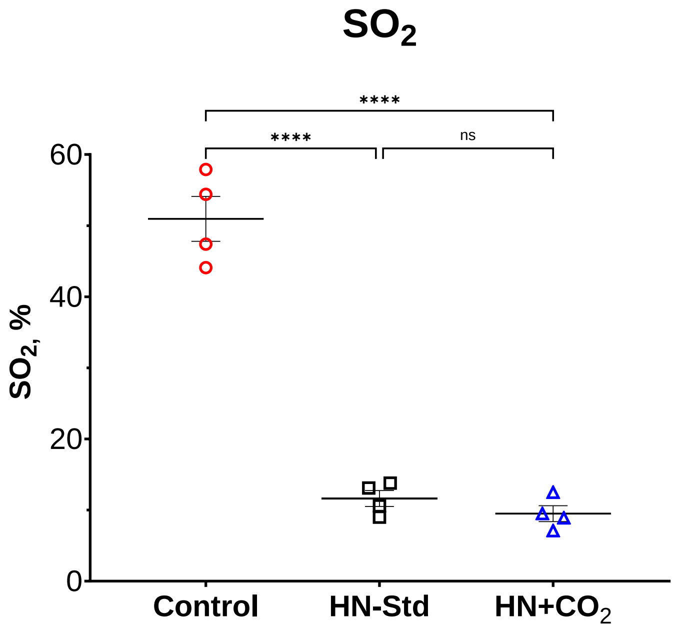

Supplementary Figure S1. Deoxygenation initial effectiveness during Hemanext process. Control, conventional RBC storage; HN-Std, standard Hemanext (hypoxic) RBC storage; and HN+CO_2, CO_2-augmented Hemanext (hypoxic) RBC storage. SO_2 data are from 4 parent superunit pools (3 subunits each) sampled at the end of the hypoxia-inducing Hemanext process. Two-way ANOVA with post-hoc Tukey's multiple comparisons test were performed. The SO_2 % in both HN-Std and HN+CO_2 were significantly lower than in Control RBCs ($p < 0.0001$). SO_2 % did not differ significantly between HN-Std and HN+CO_2 RBCs. Individual values showing with mean ± SEM. N = 4 per RBC "treatment" status.

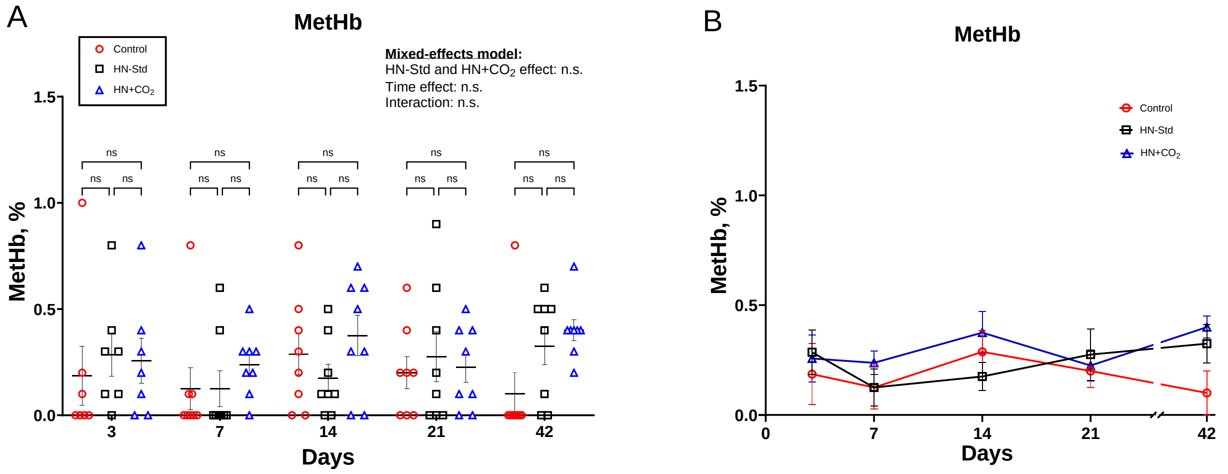

Supplementary Figure S2. Methemoglobin values were measured by cooximetry as a function of RBC storage type and storage time. No differences were statistically significantly different.

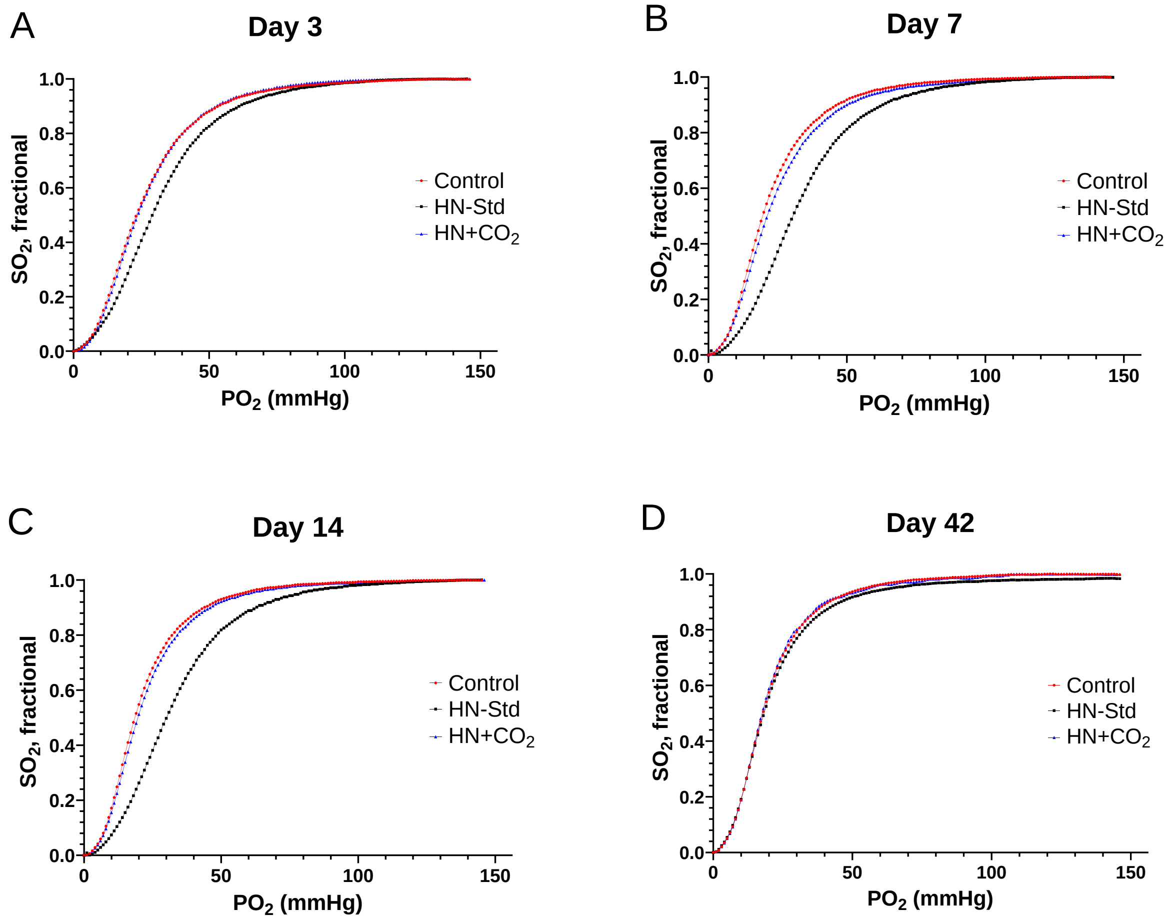

Supplementary Figure S3. Oxygen dissociation curves obtained at the indicated storage timepoints for Control, HN-Std, and HN+CO_2 RBCs from unit pool number 7 are displayed. Qualitatively similar results were seen in the corresponding RBC storage types and as a function of storage time for RBCs from unit pools 6 and 8.

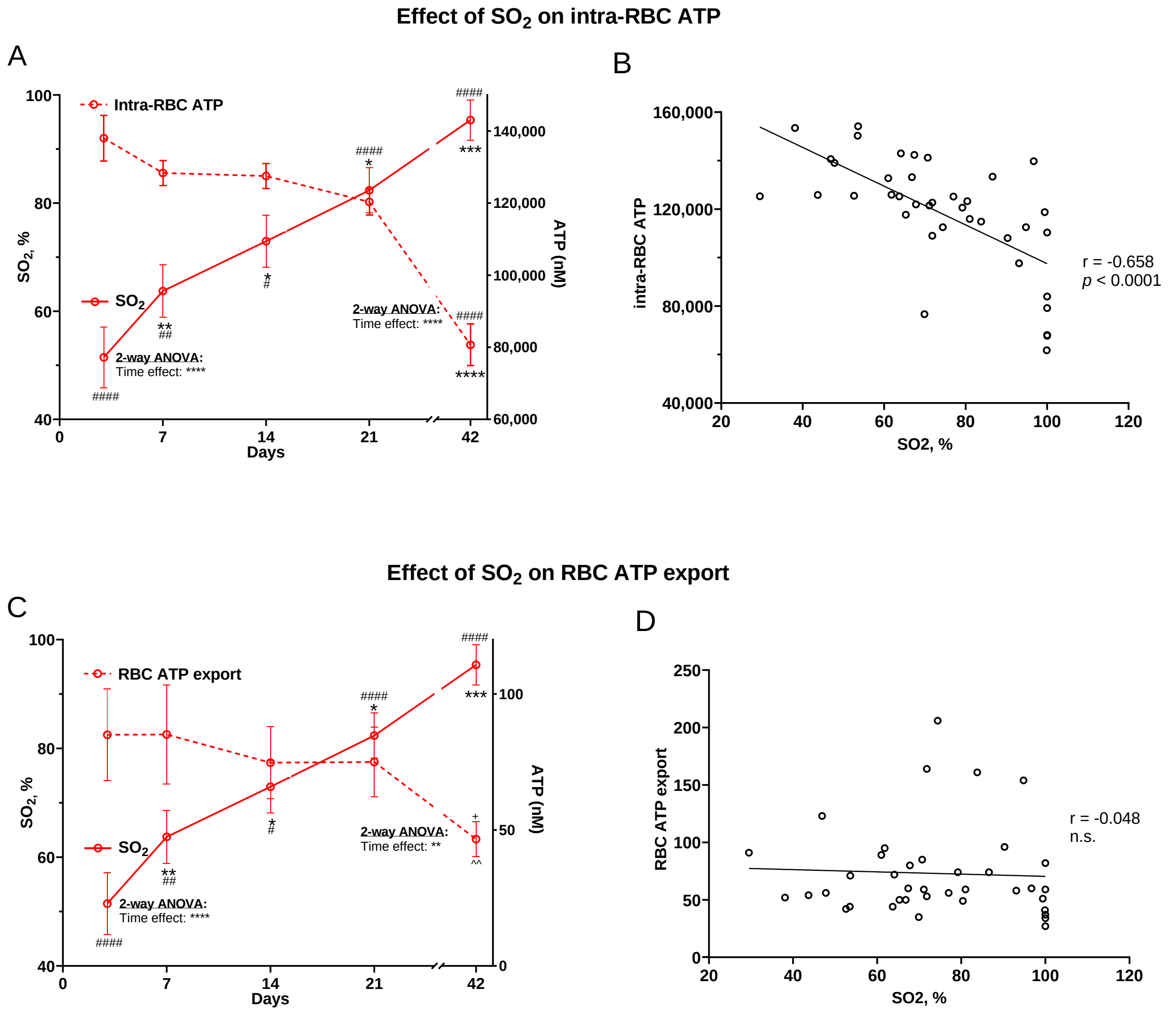

Supplementary Figure S4. Changes in SO_2 levels and either intracellular RBC ATP **(A, B)** or RBC ATP export **(C, D)** in ***conventional*** RBCs over time. Two-way repeated measures ANOVA with post-hoc Bonferroni-corrected t-testing used. *, $p < 0.05$, **, $p < 0.01$, ***, $p <$ 001, and ****, $p < 0.0001$ compared to the previous timepoint; #, $p < 0.05$, ##, $p < 0.01$, and ####, $p < 0.0001$ vs. all other timepoints; +, $p < 0.05$ vs. Day 3, and ^^, $p < 0.01$ vs. Day 7. Correlations between SO_2 and intra-RBC ATP concentration **(B)** and between SO_2 and RBC ATP export **(D)** (from all time points) are shown in the right-sided panels.

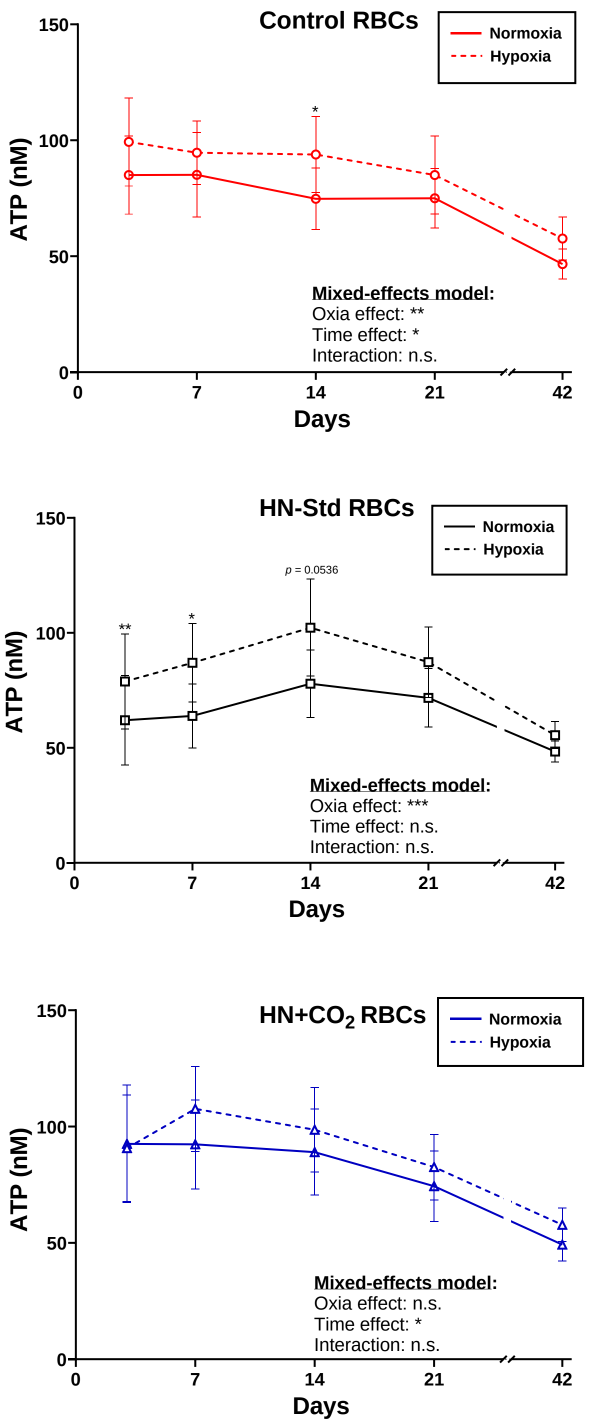

Supplementary Figure S5. RBC ATP export assayed in normoxia or hypoxia in three RBC types over time during RBC storage. *The data are identical to those in Fig. 4, but are arranged to display the results as a function of assay in Normoxia vs Hypoxia ("Oxia effect") for each RBC storage condition.* **Upper panel:** Control, conventional RBC storage; **middle panel:** HN-Std, standard Hemanext RBC storage; **lower panel:** HN+CO_2, CO_2-augmented Hemanext RBC storage. Mixed-effects model analysis results are shown for each comparison, and Sidak's post-hoc t-test was applied post-hoc. Data are mean ± SEM connected by non-fitted connector line segments. N = 8 at each time point except 7 on Day 3.

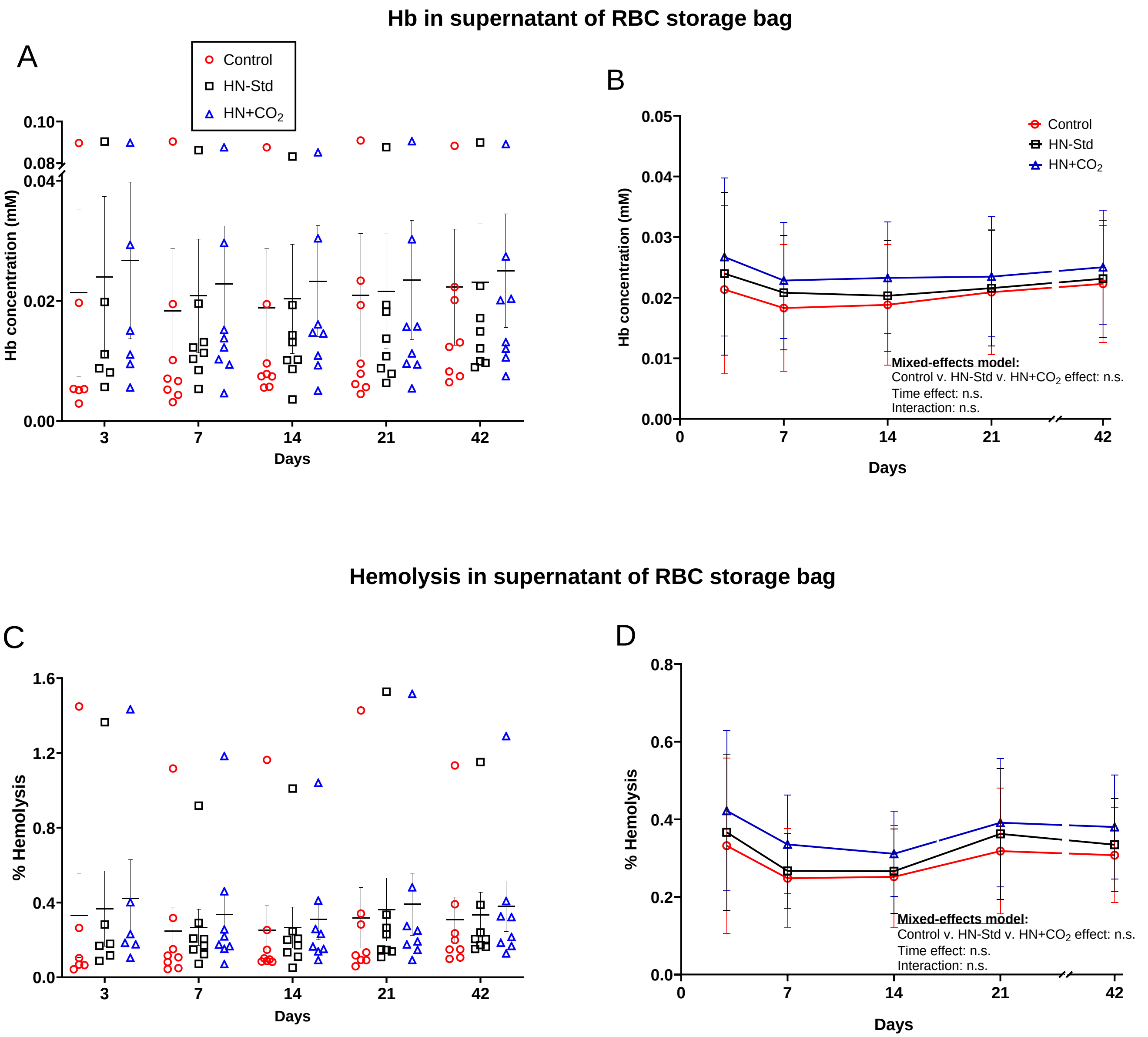

Supplementary Figure S6. Hb concentration **(A)** and hemolysis **(B)** in the supernatants of all three RBC subunits sampled over time during RBC storage. Control, conventional RBC storage; HN-Std, standard Hemanext RBC storage; and HN+CO_2, CO_2-preserved Hemanext RBC storage. No significant differences by 2-way ANOVA mixed-effects analysis with post-hoc (Tukey's t-test) at any timepoints. For visual clarity, identical data are shown in the left-sided panels (individual values, mean ± SEM), **(A, C)** and the right-sided panels **(B, D)** (mean ± SEM and connector lines). The statistical results are displayed in the right-sided panels only. N = 8 except 6 on Day 3.

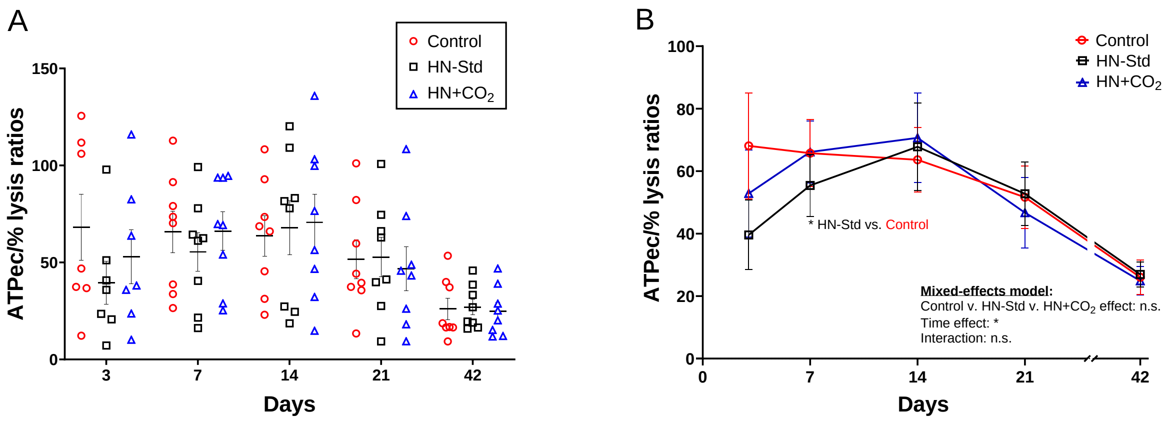

Supplementary Figure S7. Extracellular ATP in normoxia was expressed as its ratio to the % RBC lysis. Data as a function of storage time are grouped by RBC storage type. For visual clarity, identical data are shown in panel **(A)** (individual values, mean ± SEM), and panel **(B)** (mean ± SEM and connector lines). The statistical results are displayed in panel B only. N = 8 except 7 on Day 3.
